# Outcomes of different pulmonary rehabilitation protocols in patients under mechanical ventilation with difficult weaning: a retrospective cohort study

**DOI:** 10.1186/s12931-024-02866-3

**Published:** 2024-06-15

**Authors:** Shiauyee Chen, Shu-Fen Liao, Yun-Jou Lin, Chao-Ying Huang, Shu-Chuan Ho, Jer-Hwa Chang

**Affiliations:** 1 Department of Physical Medicine and Rehabilitation, Wan Fang Hospital, Taipei Medical University, Taipei, Taiwan; 2 School of Respiratory Therapy, College of Medicine, Taipei Medical University, 250 Wuxing Street, Taipei, 11031 Taiwan; 3 Department of Medical Research, Wan Fang Hospital, Taipei Medical University, Taipei, Taiwan; 4 School of Public Health, College of Public Health, Taipei Medical University, Taipei, Taiwan; 5 Institute of Epidemiology and Preventive Medicine, College of Public Health, National Taiwan University, Taipei, Taiwan; 6 Graduate Institute of Physiology, College of Medicine, National Taiwan University, Taipei, Taiwan; 7 Division of Pulmonary Medicine, Department of Internal Medicine, Shuang Ho Hospital, Taipei Medical University, New Taipei City, Taiwan; 8 Division of Pulmonary Medicine, Department of Internal Medicine, Wan Fang Hospital, Taipei Medical University, Taipei, Taiwan

**Keywords:** Mechanical ventilation with difficult weaning, Pulmonary rehabilitation, Ventilator weaning, Respiratory care center

## Abstract

**Background:**

The endeavor of liberating patients from ventilator dependence within respiratory care centers (RCCs) poses considerable challenges. Multiple factors contribute to this process, yet establishing an effective regimen for pulmonary rehabilitation (PR) remains uncertain. This retrospective study aimed to evaluate existing rehabilitation protocols, ascertain associations between clinical factors and patient outcomes, and explore the influence of these protocols on the outcomes of the patients to shape suitable rehabilitation programs.

**Methods:**

Conducted at a medical center in northern Taiwan, the retrospective study examined 320 newly admitted RCC patients between January 1, 2015, and December 31, 2017. Each patient received a tailored PR protocol, following which researchers evaluated weaning rates, RCC survival, and 3-month survival as outcome variables. Analyses scrutinized differences in baseline characteristics and prognoses among three PR protocols: protocol 1 (routine care), protocol 2 (routine care plus breathing training), and protocol 3 (routine care plus breathing and limb muscle training).

**Results:**

Among the patients, 28.75% followed protocol 1, 59.37% protocol 2, and 11.88% protocol 3. Variances in age, body-mass index, pneumonia diagnosis, do-not-resuscitate orders, Glasgow Coma Scale scores (≤ 14), and Acute Physiology and Chronic Health Evaluation II (APACHE) scores were notable across these protocols. Age, APACHE scores, and abnormal blood urea nitrogen levels (> 20 mg/dL) significantly correlated with outcomes—such as weaning, RCC survival, and 3-month survival. Elevated mean hemoglobin levels linked to increased weaning rates (*p* = 0.0065) and 3-month survival (*p* = 0.0102). Four adjusted models clarified the impact of rehabilitation protocols. Notably, the PR protocol 3 group exhibited significantly higher 3-month survival rates compared to protocol 1, with odds ratios (ORs) ranging from 3.87 to 3.97 across models. This association persisted when comparing with protocol 2, with ORs between 3.92 and 4.22.

**Conclusion:**

Our study showed that distinct PR protocols significantly affected the outcomes of ventilator-dependent patients within RCCs. The study underlines the importance of tailored rehabilitation programs and identifies key clinical factors influencing patient outcomes. Recommendations advocate prospective studies with larger cohorts to comprehensively assess PR effects on RCC patients.

**Supplementary Information:**

The online version contains supplementary material available at 10.1186/s12931-024-02866-3.

## Background

Respiratory care centers (RCCs), specialized units downstream of ICUs in Taiwan, provide effective care for patients who have repeatedly experienced ventilator weaning failure while in intensive care and have undergone prolonged mechanical ventilation [1]. In previous investigations, weaning success rates ranged from 38–70% [2–7]. Successful weaning has been attributed to several factors including age, nutritional status, comorbid conditions, muscle strength, lung mechanics, renal function, and hemoglobin (Hb) levels [1, 2, 8–10]. For adult patients on prolonged mechanical ventilation (PMV), factors associated with unsuccessful weaning include a longer duration of RCC stay, an elevated blood urea nitrogen (BUN) level, a lower modified Glasgow Coma Scale (GCS) score, and lower serum albumin and maximal inspiratory pressure (PI_max_) levels [11, 12]. Many PMV patients are discharged from the hospital but are again readmitted within one year [13]; Stoller et al’s study found that patients who were discharged from the weaning unit had a mortality rate that fell by 68% within the first 2 years [14]. There were better survival outcomes in PMV patients with the following characteristics: no comorbidities, a tracheostomy, successful weaning, and aged less than 75 years [15, 16].

Although the evidence supports that early rehabilitation can decrease ventilation duration for patients under weaning, the type and frequency of intervention are quite diverse. Prolonged weaning may impact patient’s muscular and skeletal systems. This aggregates the deconditioning even after weaning from the ventilator. Thus, early rehabilitation is important and have been reportedly related to decreased morbidity and mortality [17], disease complications [18], duration of the ICU stay, duration of the hospital stay [19, 20], the re-hospitalization rate [21], and improved in physical function [22]. Additionally, several studies show that early rehabilitation can improve weaning rate for patients with prolonged mechanical ventilation. Among the studies, treatment types are various and can be classified into conventional physical therapy, exercise-based physical therapy, neuromuscular electrical stimulation (NMES), progressive mobility, inspiratory muscle training and multi-component training programs. The training frequencies are varied from high (over 2 sessions/day), moderate (one session/day, 3–7 days/week) to low (one session/day, less than 3 days a week) [23–26].

Even with integrated care, successful weaning is difficult and related to multiple factors. The appropriate regimen of rehabilitation protocols has not been confirmed. The efficacy of pulmonary rehabilitation (PR) has been proven through random controlled trials (RCTs) and systematic meta-analyses, but implementation of PR in the real world still encounters barriers such as restricted resources or eligibility of patients. Thus, the aims of this retrospective study were to (1) review the status of rehabilitation protocols and provide information for suitable programs, (2) find associations of clinical factors with patients’ outcomes, and (3) determine the effects of rehabilitation protocols on outcomes in RCC units.

## Methods

### Study design and data sources

This was a retrospective chart review study. The study was approved by the TMU-Joint Institutional Review Board with approval no. N201612048. Patients (over 20 years of age) admitted to an RCC (a semi-ICU) from January 1, 2015 to December 31, 2017 were recruited. The RCC unit was located in a medical center in northern Taiwan (a single-center study). We gained access to the patient list and charts from our secretary department. We used traditional PR recording books and an electronic chart system for data collection [27–29].

### Setting

Patients were transferred from our medical ICU, surgical ICU, general ward, or emergent department. Patients with intermittent mandatory ventilation (IMV) could be tracheotomized or have an endotracheal tube. Patients received a multidisciplinary approach after admission. Team members included chest and critical care physicians, nurses, respiratory therapists, physical therapists, dietitians, and social workers.

### Weaning protocol

The weaning protocol for patients was discussed by team members at a bedside meeting in the morning. Patients had to meet certain criteria before attempting to wean: no hemodynamic instability; no vasopressor therapy; systolic blood pressure (SBP) of > 100 mmHg; heart rate range of 50 to 130 beats per minute; no fever; fraction of inspired oxygen of < 40%; and positive end expiratory pressure (PEEP) of < 8 cmH_2_O. Ventilatory support was gradually reduced to a level of Synchronized intermittent mandatory ventilation (SIMV), and then the level of pressure-supported ventilation (PSV) was reduced. The duration of these trials was gradually increased from 2 to 24 h. Patients were extubated when they passed the 30 min to 2-h spontaneous breathing trial and arterial blood gas (ABG) checkup.

The protocol was comprised of several steps [11, 30]. In patients with non-invasive ventilation (NIV), the time of support was gradually reduced with the oxygen supply via an oxygen mask or nasal cannula. The duration of these trials was gradually increased (beginning at 2 h and finishing at 24 h). Success in weaning was defined as 5 days of complete liberation from the ventilator.

### Intervention of rehabilitation

**Fig. 1:**
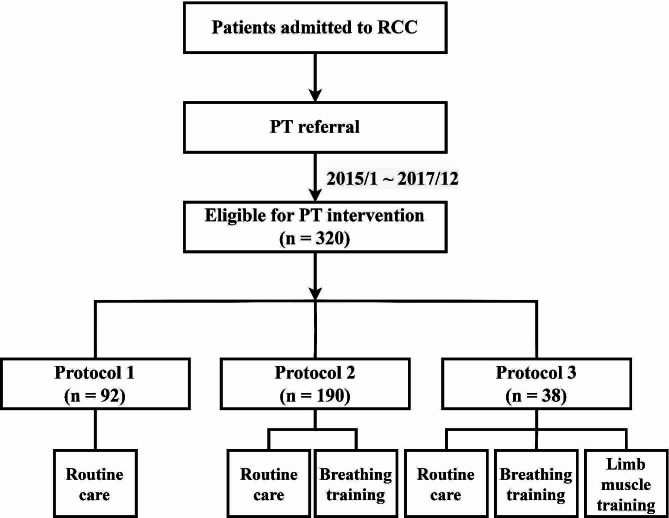
Protocols for Pulmonary Rehabilitation in the Respiratory Care Unit (RCC).

When patients were transferred to the RCC, doctors would refer the patients for physical therapy (PT). Three well-trained physical therapists with more than 3 years working in RCC conducted a rehabilitation intervention according to the algorithm of the PR protocol (Fig. 1). The PT programs were held when the patients were under one of the following situations: (1) fraction of inspired oxygen (FiO_2_) of ≥ 50% or PEEP of ≥ 8 cmH_2_O, or blood oxygen saturation level (SpO_2_) of ≤ 90%; (2) SBP of ≤ 90 or ≥ 200 mmHg; (3) respiratory rate (RR) of ≥ 35/min; (4) heart rate (HR) of ≤ 50 or ≥ 130/min; (5) a new episode of arrhythmia, e.g., ventricular premature contraction (VPC) bigeminy, bradycardia; (6) under support of a vasopressor, e.g., norepinephrine > 4 µg/min or dopamine > 5 mg/kg/min; (7) new episodes of acute myocardial infarction or chest tightness; (8) under hemodialysis (H/D); and (9) gastrointestinal (GI) bleeding [31, 32].

PT interventions provided one session a day and five sessions a week. A session lasted 15 ~ 50 min depending on the applied programs and the condition of the patient. Before the exercise intervention, PT evaluated the patients and confirmed the following items with team members: weaning plan, hemodynamics, feeding, and contraindications. The level of conscious and the ability to follow orders were also considered. PT discussed with team members and assigned patients to a specific protocol. Combinations of PT interventions depended on the patients’ conditions and PT assessments.” The pulmonary rehabilitation protocols included (1) PR protocol 1: routine care (active assisted range of motion exercise, or passive range of motion exercise, positioning, and tilt up); (2) PR protocol 2: routine care plus breathing training (deep breathing exercise and pursed-lip breathing exercise or abdominal binder or inspiratory muscle training; and (3) PR protocol 3: routine care plus breathing training and limb muscle training (ergometer bike training for lower extremities, or Thera-band resistive training for the upper extremities, or strength exercise for the lower extremities) [33, 34].

We began inspiratory muscle training when the patient was under the support of the following modes: PC/SIMV + PS or under PSV. When a patient was under the support of continuous positive airway pressure (CPAP) or oxygen support with a nasal cannula or collar, PT would hold inspiratory muscle training. The feeding schedule was coordinated with the training program. Training started 30 min to 1 h after feeding.

An abdominal binder was placed between the area of the lower rib cage and anterior superior iliac crest. The binder’s upper edge was below the costal margin so that it minimally interfered with ribcage movement. In consideration of digestion and skin allergies, the binder was released for 1 h after feeding and during the night [35, 36].

### Measurements

Primary outcomes were the weaning status, RCC survival, and 3-month survival after RCC discharge. Recorded data were as follows: demographics (age, sex, and body-mass index (BMI)), type of ventilator (IMV or NIV), rehabilitation intervention, laboratory data at RCC admission (BUN and Hb), a do-not-resuscitate (DNR) order (signed or not), a diagnosis of pneumonia, status of blood pressure at admission, Acute Physiology and Chronic Health Evaluation II (APACHE) score at RCC discharge, and GCS score at RCC entry. Patients with SBP of ≤ 90 mmHg or diastolic blood pressure (DBP) of ≤ 60 mmHg were defined as having hypotension. BUN of > 20 mg/dL and a GCS score of ≤ 14 were further applied.

### Statistical analysis

We evaluated the effects of PR protocols on prognosis including the weaning rate, RCC survival, and 3-month survival. Differences in baseline demographic characteristics and prognoses among the three PR protocols were assessed using a Chi-squared test for categorical variables and an analysis of variance (ANOVA) for continuous variables. A logistic regression model was applied to estimate odds ratios (ORs) and 95% confidence intervals (CIs) for associations between PR protocols and prognoses.

Confounding by indication bias may have been present in the study because the rehabilitation intervention may have been implemented in patients depending on their baseline characteristics, comorbidities, or clinical factors during RCC hospitalization, including age, sex, pneumonia, DNR, ventilator type, and GCS score. A propensity score, first proposed by Rosenbaum and Rubin [37], was estimated to present the probabilities of patients adopting different PR protocols. The propensity score weighting (PSW) procedure with an inverse probability of a treatment weighting approach was applied to create pseudo-populations for the three different PR protocols to account for the bias [38]. Imbalances in characteristics of indications among PR groups were well controlled in the pseudo-populations, resulting in virtual randomization of the rehabilitation intervention [39]. The pseudo-populations were used for subsequent analyses. Associations of rehabilitation with weaning, survival, and 3-month survival were examined through crude and four multiple-adjusted models. For model 1, we adjusted for sex and age only. Characteristics significantly associated with each prognosis variable in the univariate analyses were considered potential confounders and were adjusted for in multiple regression models 2 and 3. Because information on APACHE score at admission was mostly unknown in our study, we developed two different models with and without including APACHE scores. Finally, all putative confounders except for APACHE scores were adjusted for in model 4.

Statistical analyses were two-sided, and the level of significance was 0.05. All statistical analyses were performed using SAS software (vers. 9.4; SAS Institute, Cary, NC, USA).

## Results

In the study, we included 320 patients admitted to an RCC to evaluate the association between their rehabilitation protocol and their prognosis. The weaning rate and RCC survival (there was one patient with an unknown RCC survival status) were 57.50% and 89.06%, respectively. The 3-month survival status was ascertained for 236 patients (73.75% of all patients), showing a 3-month survival rate of 59.75%. Demographic characteristics of patients are presented in Table 1. All patients adopted routine care. There were 92 (28.75%), 190 (59.37%), and 38 (11.88%) patients who adopted PR protocol 1, 2, and 3, respectively. Distributions of age, BMI, a pneumonia diagnosis, having signed a DNR order, with a GCS score of ≤ 14, and an APACHE score were significantly differed among the three rehabilitation protocols. The mean age of patients receiving PR protocol 3 (73.79; SD = 13.52 years) was the youngest compared to PR protocol 1 (80.44; SD = 13.30 years) and PR protocol 2 (80.67; SD = 11.56 years). The mean BMI and APACHE score were highest for PR protocol 3 compared to PR protocol 1 and 2. There were smaller proportions of patients with pneumonia, who had signed a DNR order, and with a GCS score of ≤ 14 in the PR protocol 3 group. The proportions using IMV in the three groups were 47.83%, 57.89%, and 42.11%, respectively, with comparable distributions in the mode of ventilation. No significant differences in hypotension, BUN of > 20 mg/dL, and Hb were detected among the three PR protocols. The average duration of PT for all patients was 10.59 days (PR protocol 1: 8.00 days, PR.

**Table 1:** Demographic characteristics of subjects grouped by rehabilitation status

	PR protocol 1 (*N* = 92) *n* (%)	PR protocol 2 (*N* = 190) *n* (%)	PR protocol 3 (*N* = 38) *n* (%)	*p* value
Age, years (mean, SD)	80.44 (13.30)	80.67 (11.56)	73.79 (13.52)	**0.0065**
Sex				0.2694
Male	54 (58.70)	100 (52.63)	25 (65.79)	
Female	38 (41.30)	90 (47.37)	13 (34.21)	
Body-mass index, kg/m^2^	20.97 (3.93)	23.25 (4.92)	22.03 (5.42)	**0.0008**
Pneumonia				**0.0156**
No	13 (14.13)	41 (21.58)	14 (36.84)	
Yes	79 (85.87)	149 (78.42)	24 (63.16)	
DNR order				**0.0042**
No	23 (25.00)	67 (35.26)	21 (55.26)	
Yes	69 (75.00)	123 (64.74)	17 (44.74)	
Ventilator type				0.0990
IMV	44 (47.83)	110 (57.89)	16 (42.11)	
NIV	48 (52.17)	80 (42.11)	22 (57.89)	
GCS score of ≤ 14 at RCC admission				**< 0.0001**
No	7 (7.61)	22 (11.58)	19 (50.00)	
Yes	85 (92.39)	168 (88.42)	19 (50.00)	
APACHE score at RCC discharge	18.05 (6.68)	16.54 (5.78)	14.91 (7.50)	**0.0431**
Hypotension: SBP of ≤ 90 mmHg or DBP of ≤ 60 mmHg at RCC admission				0.7355
No	36 (39.13)	83 (43.68)	17 (44.74)	
Yes	56 (60.87)	107 (56.32)	21 (55.26)	
BUN > 20 mg/dL at RCC admission				0.4772
No	25 (28.09)	40 (21.62)	8 (21.62)	
Yes	64 (71.91)	145 (78.38)	29 (78.38)	
Hb at RCC admission	9.85 (1.42)	9.87 (1.56)	10.47 (2.13)	0.0892
Weaning				0.3256
No	39 (42.39)	85 (44.74)	12 (31.58)	
Yes	53 (57.61)	105 (55.26)	26 (68.42)	
RCC survival				**0.0475**
No	15 (16.48)	18 (9.47)	1 (2.63)	
Yes	76 (83.52)	172 (90.53)	37 (97.37)	
3-month survival				**0.0032**
No	33 (45.83)	60 (42.86)	2 (8.33)	
Yes	39 (54.17)	80 (57.14)	22 (91.67)	

There were 1, 44, and 1 missing data for the BMI, Acute Physiology and Chronic Health Evaluation II (APACHE), and hemoglobin (Hb), respectively. Data on respiratory care center (RCC) survival and 3-month survival were ascertained for 319 and 236 patients, respectively. DNR, do not resuscitate; IMV, intermittent mandatory ventilation; NIV, non-invasive ventilation; GCS, Glasgow Coma Scale; SBP, systolic blood pressure; DBP, diastolic blood pressure; BUN, blood urea nitrogen

protocol 2: **12.04** days, and PR protocol 3: **9.62** days).

### Propensity score-weighted cohort

In order to reduce confounding by indication bias due to an imbalance of rehabilitation protocol assignments, we performed a propensity score-weighting approach in this study. Age, sex, pneumonia, DNR, ventilator type, and GCS were used to calculate propensity scores. After the weighting procedure, the weighted cohort comprised 315.28 patients in total, with 89.82, 189.84, and 35.62 patients in PR protocols 1, 2, and 3, respectively (Table 2). All variables included in the propensity score were balanced in the three rehabilitation protocols among the weighted cohort.

**Table 2:** Distributions of rehabilitation and demographic characteristics among propensity score-weighted cohorts

	PR protocol 1 (*N* = 89.82) *n* (%)	PR protocol 2 (*N* = 189.84) *n* (%)	PR protocol 3 (*N* = 35.62) *n* (%)	*p* value
Age, years (mean, SD)	80.33 (12.74)	79.93 (11.93)	78.71 (11.48)	0.7947
Sex				0.5706
Female	36.65 (40.80)	83.71 (44.09)	12.44 (34.93)	
Male	53.18 (59.20)	106.14 (55.91)	23.18 (65.07)	
Pneumonia				0.8305
No	17.71 (19.71)	39.42 (20.76)	8.76 (24.59)	
Yes	72.12 (80.29)	150.42 (79.24)	26.86 (75.41)	
DNR				0.6976
No	29.77 (33.14)	64.84 (34.15)	14.56 (40.87)	
Yes	60.06 (66.86)	125.01 (65.85)	21.06 (59.13)	
Ventilator type				0.9450
IMV	49.34 (54.93)	100.79 (53.09)	18.57 (52.14)	
NIV	40.48 (45.07)	89.06 (46.91)	17.05 (47.86)	
GCS ≤ 14 at RCC admission				0.7379
No	10.84 (12.07)	28.23 (14.87)	6.01 (16.87)	
Yes	78.99 (87.93)	161.61 (85.13)	29.61 (83.13)	

^**&**^ Propensity score by age, sex, pneumonia, do not resuscitate (DNR), ventilator type, and Glasgow Coma Scale (GCS). IMV, intermittent mandatory ventilation; NIV, non-invasive ventilation; RCC, respiratory care center

### Rehabilitation, demographic characteristics, and prognosis

Distributions of rehabilitation and demographic characteristics of weaning, survival, and 3-month survival among the weighted cohort are demonstrated in Table 3. Rehabilitation showed a borderline significant effect on 3-month survival (*p* = 0.0554), but not on the weaning rate or RCC survival. We found that 58.65%, 56.83%, and 84.61% of patients adopting PR protocol 1, 2, and 3 were alive at 3 months, respectively. Age, APACHE score, and an abnormal BUN level (> 20 mg/dL) were significantly associated with the prognosis, including weaning, RCC survival, and 3-month survival. Signing a DNR order and ventilator type were also critical factors related to RCC survival and 3-month survival. Patients diagnosed with pneumonia or detected with hypotension presented a poorer prognosis than patients without the corresponding disease (pneumonia: 87.55% vs. 97.16% for RCC survival; hypotension: 52.32% vs. 70.69% for 3-month survival). A higher mean Hb was associated with a higher weaning rate (*p* = 0.0065) and 3-month survival (*p* = 0.0102).

**Table 3:** Distributions of rehabilitation and demographic characteristics on weaning, survival, and 3-month survival among propensity score-weighted cohorts

	Weaning (*N* = 315.28)			RCC survival (*N* = 313.55)			3-month survival (*N* = 230.39)		
	No *n* (%)	Yes *n* (%)	*p* value	No *n* (%)	Yes *n* (%)	*p* value	No *n* (%)	Yes *n* (%)	*p* value
Rehabilitation			0.9378			0.2697			0.0554
PR protocol 1	36.61 (40.76)	53.22 (59.24)		12.66 (14.37)	75.43 (85.63)		28.65 (41.35)	40.64 (58.65)	
PR protocol 2	81.66 (43.02)	108.18 (56.98)		18.38 (9.68)	171.46 (90.32)		60.72 (43.17)	79.95 (56.83)	
PR protocol 3	14.97 (42.04)	20.65 (57.96)		1.84 (5.17)	33.78 (94.83)		3.14 (15.39)	17.29 (84.61)	
Age, years	81.77 (11.21)	78.54 (12.55)	**0.0186**	83.96 (7.65)	79.50 (12.42)	**0.0050**	82.72 (9.10)	78.06 (13.59)	**0.0021**
Sex			0.2022			0.2344			0.7919
Female	61.64 (46.42)	71.15 (53.58)		17.11 (12.89)	115.68 (87.11)		39.82 (41.16)	56.93 (58.84)	
Male	71.60 (39.23)	110.89 (60.77)		15.77 (8.72)	164.99 (91.28)		52.70 (39.43)	80.95 (60.57)	
Body-mass index, kg/m^2^	22.70 (4.45)	22.41 (5.15)	0.5927	22.07 (4.42)	22.61 (4.92)	0.5478	22.16 (4.40)	22.93 (5.33)	0.2337
Pneumonia			0.8337			**0.0250**			0.2679
No	27.09 (41.12)	38.79 (58.88)		1.82 (2.84)	62.33 (97.16)		14.10 (32.70)	29.03 (67.30)	
Yes	106.15 (42.56)	143.25 (57.44)		31.06 (12.45)	218.34 (87.55)		78.41 (41.87)	108.85 (58.13)	
Do not resuscitate (DNR) order			0.1104			**0.0017**			**0.0003**
No	39.47 (36.16)	69.69 (63.84)		3.16 (2.95)	104.26 (97.05)		18.68 (23.98)	59.22 (76.02)	
Yes	93.77 (45.49)	112.35 (54.51)		29.72 (14.42)	176.41 (85.58)		73.83 (48.42)	78.65 (51.58)	
Ventilator type			0.1652			**0.0005**			**0.0308**
IMV	77.37 (45.86)	91.34 (54.14)		8.03 (4.81)	158.94 (95.19)		39.58 (33.38)	79.00 (66.62)	
NIV	55.88 (38.12)	90.71 (61.88)		24.86 (16.96)	121.73 (83.04)		52.93 (47.34)	58.88 (52.66)	
GCS score of ≤ 14 at RCC admission			0.1347			0.4665			0.1966
No	14.46 (32.07)	30.62 (67.93)		6.11 (13.56)	38.96 (86.44)		9.30 (29.65)	22.07 (70.35)	
Yes	118.79 (43.96)	151.43 (56.04)		26.77 (9.97)	241.71 (90.03)		83.21 (41.81)	115.80 (58.19)	
APACHE score at RCC discharge	18.10 (7.11)	15.79 (5.64)	**0.0045**	19.71 (7.91)	16.43 (6.15)	**0.0139**	18.19 (6.62)	15.96 (5.72)	**0.0136**
Hypotension: SBP ≤ 90 mmHg or DBP ≤ 60 mmHg at RCC admission			0.3742			0.1790			**0.0051**
No	53.64 (39.42)	82.42 (60.58)		10.48 (7.80)	123.85 (92.20)		27.67 (29.31)	66.74 (70.69)	
Yes	79.60 (44.42)	99.62 (55.58)		22.40 (12.50)	156.82 (87.50)		64.84 (47.68)	71.14 (52.32)	
BUN of > 20 mg/dL^$^ at RCC admission			**0.0109**			**0.0318**			**< 0.0001**
No	21.54 (29.30)	51.97 (70.70)		2.47 (3.44)	69.30 (96.56)		7.92 (15.90)	41.89 (84.10)	
Yes	107.73 (46.11)	125.92 (53.89)		28.46 (12.18)	205.19 (87.82)		81.77 (47.18)	91.55 (52.82)	
Hb at RCC admission	9.67 (1.33)	10.13 (1.68)	**0.0065**	9.53 (1.31)	9.98 (1.58)	0.1177	9.64 (1.37)	10.17 (1.72)	**0.0102**

^**&**^ Propensity score by age, sex, pneumonia, DNR, ventilator type, and Glasgow Coma Scale (GCS) score

^$^ Missing blood urea nitrogen (BUN) data: *n* = 8.13

PR, pulmonary rehabilitation; IMV, intermittent mandatory ventilation; NIV, non-invasive ventilation; RCC, respiratory care center; APACHE, Acute Physiology and Chronic Health Evaluation II; SBP, systolic blood pressure; DBP, diastolic blood pressure; Hb, hemoglobin

### Effects of rehabilitation protocols on the prognosis

To investigate associations of rehabilitation protocols with weaning, survival, and 3-month survival, a univariate analysis and several adjusted models were performed in the study (Table 4). In the univariate analysis, there were no significant associations between rehabilitation protocols and weaning or RCC survival. Patients adopting PR protocol 3 (OR: 3.87; 95% CI: 1.08–13.90; *p* = 0.0378) showed a better 3-month survival compared to those adopting PR protocol 1, but the beneficial effect was not presented for the PR protocol 2 group.

**Table 4:** Associations of rehabilitation protocols with weaning, survival, and 3-month survival after propensity score weighting

Weaning	Crude		Model 1: adjusted for sex and age		Model 2: adjusted for sex, age, BUN, and Hb		Model 3: adjusted for sex, age, BUN, Hb, and APACHE		Model 4: adjusted for sex, age, DNR, ventilator type, hypotension, BUN, pneumonia, GCS, and Hb	
	OR (95% CI)	*p* value	OR (95% CI)	*p* value	OR (95% CI)	*p* value	OR (95% CI)	*p* value	OR (95% CI)	*p* value
Rehabilitation										
PR protocol 1	Ref.		Ref.		Ref.		Ref.		Ref.	
PR protocol 2	0.91 (0.55, 1.51)	0.7190	0.91 (0.54, 1.51)	0.7080	0.95 (0.56, 1.62)	0.8481	0.82 (0.45, 1.49)	0.5131	0.93 (0.54, 1.59)	0.7840
PR protocol 3	0.95 (0.43, 2.07)	0.8944	0.90 (0.41, 1.97)	0.7841	0.85 (0.38, 1.91)	0.6917	0.61 (0.25, 1.49)	0.2806	0.79 (0.35, 1.79)	0.5722
**RCC survival**	Crude		Model 1: adjusted for sex and age		Model 2: adjusted for sex, age, DNR, pneumonia, ventilator type, and BUN		Model 3: adjusted for sex, age, DNR, pneumonia, ventilator type, BUN, and APACHE		Model 4: adjusted for sex, age, DNR, ventilator type, hypotension, BUN, pneumonia, GCS, and Hb	
	OR (95% CI)	*p* value	OR (95% CI)	*p* value	OR (95% CI)	*p* value	OR (95% CI)	*p* value	OR (95% CI)	*p* value
Rehabilitation										
PR protocol 1	Ref.		Ref.		Ref.		Ref.		Ref.	
PR protocol 2	1.57 (0.73, 3.34)	0.2467	1.56 (0.72, 3.35)	0.2570	1.83 (0.80, 4.23)	0.1552	1.35 (0.49, 3.71)	0.5572	1.79 (0.77, 4.20)	0.1776
PR protocol 3	3.08 (0.63, 15.01)	0.1642	2.78 (0.57, 13.71)	0.2081	2.78 (0.53, 14.72)	0.2291	2.00 (0.34, 11.92)	0.4452	2.29 (0.41, 12.75)	0.3455
**3-month survival**	Crude		Model 1: adjusted for sex and age		Model 2: adjusted for sex, age, DNR, ventilator type, hypotension, BUN, and Hb		Model 3: adjusted for sex, age, DNR, ventilator type, hypotension, BUN, Hb, and APACHE		Model 4: adjusted for sex, age, DNR, ventilator type, hypotension, BUN, pneumonia, GCS, and Hb	
	OR (95% CI)	*p* value	OR (95% CI)	*p* value	OR (95% CI)	*p*- value	OR (95% CI)	*p* value	OR (95% CI)	*p* value
Rehabilitation										
PR protocol 1	Ref.		Ref.		Ref.		Ref.		Ref.	
PR protocol 2	0.93 (0.52, 1.65)^$^	0.7997	0.92 (0.51, 1.66)^#^	0.7852	1.01 (0.52, 1.95)^$$^	0.9736	0.89 (0.42, 1.87)	0.7513	1.08 (0.57, 2.06)	0.9807
PR protocol 3	3.87 (1.08, 13.90)^$^	**0.0378**	3.89 (1.06, 14.23)^#^	**0.0400**	3.97 (1.02, 15.43)^$$^	**0.0469**	3.13 (0.74, 13.24)	0.1205	4.76 (1.22, 18.50)	0.0525

^**&**^ Propensity score by age, sex, pneumonia, do not resuscitate (DNR), ventilator type, and Glasgow Coma Scale (GCS); ^$^ OR = 4.17 (1.22, 14.31), *p* = 0.0230 (PR protocol 2 as the reference); ^#^ OR = 4.22 (1.21, 14.74), *p* = 0.0240 (PR protocol 2 as the reference); ^$$^ OR = 3.92 (1.07, 14.39), *p* = 0.0394 (PR protocol 2 as the reference)

OR, odds ratio; CI confidence interval; Ref., reference; PR, pulmonary rehabilitation; BUN, blood urea nitrogen; Hb, hemoglobin; APACHE, Acute Physiology and Chronic Health Evaluation II.

We further applied four adjusted models to clarify the effect of rehabilitation protocols, including model 1 (adjusted for sex and age), model 2 (adjusted for all variables except the APACHE score that reached statistical significance in the univariate analysis), model 3 (adjusted for all variables that reached statistical significance in the univariate analysis), and model 4 (adjusted for sex, age, DNR, ventilator type, hypotension, BUN, pneumonia, GCS, and Hb). Results were similar to those shown in the univariate analysis (Table 4). PR protocol 2 was not significantly associated with weaning, survival, or 3-month survival, compared to PR protocol 1. According to models 1 and 2, 3-month survival in the PR protocol 3 group was significantly higher than that in the PR protocol 1 group, with ORs of 3.89 (95% CI: 1.06–14.23, *p* = 0.04) and 3.97 (95% CI: 1.02–15.43, *p* = 0.0469), respectively. The significant association remained when taking the PR protocol 2 group as the reference, with ORs of 3-month survival in the PR protocol 3 group of 4.17 (95% CI: 1.22–14.31, *p* = 0.0230), 4.22 (95% CI: 1.21–14.74, *p* = 0.0240), and 3.92 (95% CI: 1.07–14.39, *p* = 0.0394) under the univariate analysis, model 1, and model 2, respectively. The beneficial effect of PR protocol 3 (compared to PR protocol 1) became borderline significant or non-significant under models 4 and 3, possibly because of insufficient power in the analyses.

## Discussion

This retrospective chart review study showed that different pulmonary rehabilitation protocols were associated with 3-month survival after discharge from an RCC, but not the weaning status or survival in RCC hospitalization. Patients who received routine care plus respiratory muscle and limb muscle training had an OR of 3.87 in the crude model compared to routine care for the outcome of 3-month survival (with a survival rate of 84.61%).

In a retrospective analysis over 10 years [40], 180 tracheostomized patients received PR programs during weaning at a semi-ICU. The individual rehabilitation programs (IRPs) included intensive physiotherapy (peripheral and respiratory muscle reconditioning, airway secretion, and breathing exercises). The PR programs were executed twice a day for 1 h each time. The programs were adjusted according to a patient’s clinical stability. Results showed that the level of comorbidities (Cumulative Illness Rating Scale) was associated with weaning outcomes. The weaning rate for patients was 66.1%. In our study, our patients included those with an endotracheal tube (*n* = 76), those tracheostomized (*n* = 94), and those with NIV (*n* = 150), and the weaning rate for all subjects was 57.50%. The PR programs in our study were also tailored by our team members, but only once daily. Our programs provided three main components: routine care, breathing training, and limb muscle training. Not all of the patients received all components. Our study also found that the APACHE score was significantly associated with weaning outcomes. Since the APACHE score is a general measure of disease severity, our study had similar findings to a study by Costi et al. [34] Under implementation of PR, the weaning outcome was still influenced by the level of severity.

Keng et al. [41] found that the post-rehabilitation functional status was independently associated with the weaning success, 3-month survival after RCC discharge, and hospital survival. The de Morton Morbidity Index (DEMMI) was selected as a tool to measure the responsiveness to PR rather than the acceptance of PR itself. Patients in this study were under prolonged mechanical ventilation and received standard sessions of the training program. Due to concern with patient tolerance and hemodynamic stability, we assigned patients to appropriate regimens in our study after discussion with team members. Maybe that resulted in a lower intensity or duration which would have impacted weaning and RCC survival. Patient who were rehabilitation responders (post-rehab DEMMI of ≥ 20) had significantly higher weaning success and survival at hospital discharge and at 3 months after RCC discharge regardless of the pre-rehab DEMMI score. That supports the importance of PR protocols in the unit focused on weaning. Our study revealed that patients who adopted PR protocol 3 showed a better 3-month survival compared to those who adopted PR protocol 1 (OR: 3.87). Patients in PR protocol 3 received limb muscle training, which should have been beneficial for functional recovery. For those poor-responders to rehabilitation, clinical outcomes may have been limited by other factors. Results of our study found that age, APACHE score, BUN level, signing a DNR order, ventilator type, a pneumonia diagnosis, hypotension, and Hb level were associated with weaning, RCC survival, or 3-month survival.

Controversies in outcomes may depend on the subject eligibility criteria, content of the programs, and organization’s facilities [22, 23, 25]. Verceles’s study applied multimodal rehabilitation programs (MRP) for patients with PMV and ICU acquired weakness. Results showed that patients with usual care (UC) plus multimodal training programs (functional training: bed dependent to chair dependent or ambulatory programs) had greater weaning success, and more were discharged to home [23]. Our study had similar finding with this study. Patients under PR protocol 3 with limb muscle training had a higher 3-month survival rate (84.61%). The ages of subjects in Verceles’s study were 63.1 vs. 57.1 years (MRP + UC vs. UC). The pre-admission Barthel index scores were 90 vs. 96 (indicating a higher functional status). In case of a higher functional status before admission, subjects could tolerate intensive and functional mobility, strength, and endurance training [23]. The population in our subjects were older (79.79 ± 12.48 years). Since 85% of our subjects had a GCS score of ≤ 14, we could only apply routine care plus breathing training (abdominal binder or inspiratory muscle training) instead of limb muscle training or functional approaches.

We also discovered that younger patients were more quickly weaned from the ventilator, and maintained better RCC survival and 3-month survival. Past studies found that age had an independent effect on the result of patients with mechanical ventilation, such as ventilator dependence [42], complications, the duration of mechanical ventilation, and hospital stay [43].

Yang et al. found that APACHE II scores and serum albumin concentrations were the best weaning predictors in patients with pneumonia [1]. In our 78.75% of patients with pneumonia, we found that APACHE scores and serum BUN levels at RCC admission were significantly related to successful weaning, RCC survival, and 3-month survival. The mean APACHE score of reports of successfully weaned pneumonia patients was 16.9, while our mean APACHE score of 16.77 was consistent with those of Yang et al. Malnutrition may be an important factor in an RCC patient’s ability to be weaned from mechanical ventilation. Higher Hb levels above 10.2 g/dL seemed to increase the successful weaning rate [1]. In our study, we also found that higher hemoglobin levels above 10.13 g/dL seemed to increase the successful weaning rate and above 10.17 g/dL to increase 3-month survival.

### Strengths and limitations

The strengths of our study included (1) offering different protocols for subjects, (2) defining contraindications and criteria to cease training, and (3) using propensity score weighting for the analysis to reduce confounding by indication bias due to imbalanced distributions in characteristics among the three different PR protocols. Results provide greater evidence because the study design was approximately like that of an RCT [38]. Limitations of our study included (1) a lack of pulmonary function measurement and a measure of the functional status before entry and discharge from the RCC such as Functional Independence Measure (FIM) or DEMMI. Pulmonary function has been considered a crucial variable for survival rates but it cannot be well controlled in the study. (2) This was a single study of a retrospective nature, which only reflected the experience in a single regional weaning unit, and so cannot be generalized to facilities in other regions. (3) Unavoidable data loss occurred for some parameters during clinical practice such as APACHE scores (missing for 44 patients) and 3-month survival data. This may have reduced the statistical power and resulted in an inability to replicate the significant association between PR protocols and 3-month survival in adjusted model 2 when APACHE scores were additionally included (model 3).

As to loss of data for 3-month survival, in the period of cohort studies, we could not easily obtain all of the outpatient data after discharge if patients did not return to our hospital. To evaluate the impact on our findings of data loss after discharge, we conducted a comparison of baseline characteristics and PR protocols between 3-month responders and non-responders among RCC survivors (supplementary Table 1). Similar distributions of the baseline characteristics in the two groups revealed the data loss was at random and the effect of PR protocols on 3-month survival obtained from the study was an under-estimate to true effect. In addition, we compared the 3-month survival according to the respiratory care ward (RCW) admission status after discharge from hospital among 3-month responders (supplementary Table 2) and found non-significant distribution in between.

## Conclusion

From the data in our stduy, PR protocol in individual patient could be beneficial in the outcomes when we take associated factors into consideration. Early implementation and appropriate protocols are important for the patient with difficult weaning status. Ddifferent rehabilitation protocols, especially the protocol 3 including all three components of pulmonary rehabilitation programs as basic elements, breathing exercise and limb muscle training mostly benefit for patients under ventilator dependence, even for the long-termed survival rate. This might support an integrating rehabilitation program in future clinical field. However, being a retrospective study in a single weaning hospital unit as its inevitable limitations of our studies, it seems reasonable to conduct a prospective program to validate the significant effect of incorporated rehabilitation programs. Maybe in the future, these evidences would help clinical professionals for conducting rehabilitation programs for these patient population.

### Electronic supplementary material

Below is the link to the electronic supplementary material.

Supplementary Material 1

Supplementary Material 2

## Data Availability

No datasets were generated or analysed during the current study.

## Abbreviations

RCC: Respiratory care centers
PR: Pulmonary rehabilitation
APACHE: Acute Physiology and Chronic Health Evaluation II
PMV: Prolonged mechanical ventilation
BUN: Blood urea nitrogen
GCS: Glasgow Coma Scale
MRP: Multimodal rehabilitation program
IMV: Intermittent mandatory ventilation
PEEP: Positive end expiratory pressure
SIMV: Synchronized intermittent mandatory ventilation
PSV: Pressure supported ventilation
ABG: Arterial blood gas
NIV: Not-invasive ventilation
PT: Physical therapy
FiO_2_: Fraction of inspired oxygen
CPAP: Continuous positive airway pressure
DNR: Do-not-resuscitate (DNR)
PSW: Propensity score weighting (PSW)
