# Supplementary material for: Outcomes of different pulmonary rehabilitation protocols in patients under mechanical ventilation with difficult weaning: a retrospective cohort study
Source: Respir Res. 2024 Jun 15;25:243. doi: 10.1186/s12931-024-02866-3 (PMC11180404; doi:10.1186/s12931-024-02866-3)
Supplement: Supplementary file 1 — Supplementary Material 1 [file 12931_2024_2866_MOESM1_ESM.docx]

Supplementary table 1. The comparison of baseline characteristics and PR protocols between 3-month responders and non-responders among 285 RCC survivors

|  | 3-month non-responders  (*N*=83)  *n* (%) | 3-month responders  (*N*=202)  *n* (%) | *p*-value |
| --- | --- | --- | --- |
| Age, years (mean, SD) | 80.13 (11.97) | 78.87 (13.15) | 0.4492 |
| Sex |  |  | 0.8289 |
| Male | 48 (57.83) | 114 (56.44) |  |
| Female | 35 (42.17) | 88 (43.56) |  |
| BMI, kg/m^2^ | 22.34 (4.66) | 22.60 (4.98) | 0.6839 |
| Pneumonia |  |  | 0.2060 |
| No | 23 (27.71) | 42 (20.79) |  |
| Yes | 60 (72.29) | 160 (79.21) |  |
| Do not resuscitate (DNR) order |  |  | 0.5606 |
| No | 29 (34.94) | 78 (38.61) |  |
| Yes | 54 (65.06) | 124 (61.39) |  |
| Ventilator type |  |  | 0.4131 |
| IMV | 50 (60.24) | 111 (54.95) |  |
| NIV | 33 (39.76) | 91 (45.05) |  |
| GCS≤14 at RCC admission |  |  | 0.5905 |
| No | 14 (16.87) | 29 (14.36) |  |
| Yes | 69 (83.13) | 173 (85.64) |  |
| APACHE at RCC discharge |  |  |  |
| Hypotension: SBP≤90 mmHg or DBP≤60 mmHg at RCC admission |  |  | 0.8154 |
| No | 37 (44.58) | 87 (43.07) |  |
| Yes | 46 (55.42) | 115 (56.93) |  |
| BUN>20 mg/dL at RCC admission |  |  | 0.6819 |
| No | 22 (26.83) | 48 (24.49) |  |
| Yes | 60 (73.17) | 148 (75.510 |  |
| Hb at RCC admission | 9.92 (1.50) | 10.01 (1.69) | 0.6929 |
| Rehabilitation |  |  | 0.3702 |
| PR protocol 1 | 19 (22.89) | 57 (28.22) |  |
| PR protocol 2 | 50 (60.24) | 122 (60.40) |  |
| PR protocol 3 | 14 (16.87) | 23 (11.39) |  |
