# Supplementary material for: Outcomes of different pulmonary rehabilitation protocols in patients under mechanical ventilation with difficult weaning: a retrospective cohort study
Source: Respir Res. 2024 Jun 15;25:243. doi: 10.1186/s12931-024-02866-3 (PMC11180404; doi:10.1186/s12931-024-02866-3)
Supplement: Supplementary file 2 — Supplementary Material 2 [file 12931_2024_2866_MOESM2_ESM.docx]

Supplementary table 2. The 3-month survival by respiratory care ward admission status after discharge among 3-month responders (N=202).

|  | **3-month survival** | | |
| --- | --- | --- | --- |
|  | No  *n* (%) | Yes  *n* (%) | *p*-value |
| Admission to respiratory care ward |  |  | 0.5939 |
| No | 59 (30.57) | 134 (69.43) |  |
| Yes | 2 (22.22) | 7 (77.78) |  |
